# Congenital Hypogonadotrophic Hypogonadism: Minipuberty and the Case for Neonatal Diagnosis

**DOI:** 10.3389/fendo.2019.00097

**Published:** 2019-02-21

**Authors:** Du Soon Swee, Richard Quinton

**Affiliations:** ^1^Department of Endocrinology, Royal Victoria Infirmary, Newcastle upon Tyne Hospitals NHS Foundation Trust, Newcastle upon Tyne, United Kingdom; ^2^Department of Endocrinology, Singapore General Hospital, Singapore, Singapore; ^3^Institute of Genetic Medicine, University of Newcastle upon Tyne, Newcastle upon Tyne, United Kingdom

**Keywords:** congenital hypogonadotropic hypogonadism, kallmann syndrome, puberty delay, minipuberty of infancy, infertility–male, cryptorchidism, gonadotrophin releasing hormone deficiency

## Abstract

Congenital hypogonadotrophic hypogonadism (CHH) is a rare but important etiology of pubertal failure and infertility, resulting from impaired gonadotrophin-releasing hormone secretion or action. Despite the availability of effective hormonal therapies, the majority of men with CHH experience unsatisfactory outcomes, including chronic psychosocial and reproductive sequelae. Early detection and timely interventions are crucial to address the gaps in medical care and improve the outlook for these patients. In this paper, we review the clinical implications of missing minipuberty in CHH and therapeutic strategies that can modify the course of disease, as well as explore a targeted approach to identifying affected male infants by integrating clinical and biochemical data in the early postnatal months.

## Introduction

Congenital hypogonadotrophic hypogonadism (CHH) is a rare genetic condition characterized by reproductive disorder due to deficiency in secretion or action of gonadotrophin-releasing hormone (GnRH). It is traditionally considered to be a male-predominant condition, with a male: female gender ratio 3.6: 1 that remains unexplained (1). The major clinical consequences of CHH are pubertal failure and infertility.

Although generally considered a rare disorder, accurate determination of the prevalence of CHH has not been possible because of scarce literature. Based on a French study of potential military conscripts who attended medical examination (2) and, more recently, a retrospective study of nationwide hospital records in Finland (3), (both of which were methodologically prone to under-ascertainment), male CHH prevalence of 1 in 4,415–15,000 is currently estimated.

The genetic defects underpinning CHH broadly fall into two principle groups, comprising (a) those causing neurodevelopmental defects of GnRH neuron migration frequently associated with non-reproductive defects, particularly anosmia/hyposmia from olfactory axon misrouting (i.e., Kallmann syndrome–KS), and (b) those causing pure neuroendocrine impairment of GnRH secretion or action (normosmic CHH). Belying this apparently simple dichotomy is the huge diversity of genetic mutations, with over 30 gene loci implicated to date, despite nearly half of CHH cases remaining unaccounted for. Moreover, some genes are implicated in both normosmic CHH and KS (4). This complexity is also reflected in the various modes of transmission possible, including oligogenicity as well as all forms of classical Mendelian inheritance (4, 5).

Furthermore, CHH is phenotypically heterogeneous. Besides the variable association with non-reproductive features, such as deafness, synkinesis (mirror movements), renal agenesis, digital and dental anomalies, and clefting, reproductive manifestation range from absent puberty, pubertal arrest, to even spontaneous reversal of hypogonadism in a small minority (5). CHH males frequently present with cryptorchidism and/or micropenis, which are important features of severe fetal-infancy GnRH deficiency (absent minipuberty) (6). However, patient experiences indicate that these early presentations only rarely signpost timely disease recognition and treatment-initiation in later life.

Despite medical advances in genetics, diagnostics and treatment, health outcomes of CHH men remain disappointing, with a significant proportion bearing the long-term consequences of suboptimal care (7). In this chapter, we will review the factors contributing to poor outcomes of men with CHH, and the strategies that can improve the quality of life and fertility potential, with special focus on harnessing the window of minipuberty for early diagnosis, and intervention.

## Delayed Diagnosis and Treatment

Delayed puberty is the main mode of presentation in CHH males. Approximately 2/3 of CHH males adolescents do not show any sign of spontaneous puberty at >17 years of age (testis volume <4 mL), with the remainder exhibiting arrested early puberty (8).

Unfortunately, CHH is biochemically indistinguishable from constitutional delay in growth and puberty (CDGP), the latter accounting for up to 65% of delayed puberty in younger teenage boys (9), but obviously declining precipitously with advancing age at presentation. Both conditions are characterized by low sex steroids in association with low or inappropriately normal follicle-stimulating hormone (FSH) and luteinizing hormone (LH) levels. In terms of their height development, the baseline height SD scores and growth velocities do not appear to differ significantly between CDGP and CHH adolescents, unlike in functional hypogonadotrophic hypogonadism where there is a tendency for lower height SD scores and reduced growth velocity of <3 cm/year (9, 10). It is however worth noting that progressive reduction in height SDs has been observed in a proportion of CDGP boys during the pre-pubertal years, with final attained height falling short of their genetic potential (11). On the other hand, preservation of height relative to parental height in CHH patients has been suggested (12). Hence, careful interrogation of the growth history can yield useful information.

Although a variety of stimulation tests (such as GnRH stimulation test and hCG test) and, recently, inhibin B (I_B_) concentrations (marker of Sertoli cell function), have been proposed, there is still lack of consensus on the “gold standard” test to reliably distinguish them (13). Hence, making a diagnosis of CHH remains a challenging task and clinicians frequently default to the classic dogma of expectant management, i.e., allowing adolescents enough time for those with CDGP to undergo spontaneous initiation of puberty, as a means to identify those with CHH (14). It is noteworthy that in a recent large single center retrospective series, the combination of testicular volume and basal I_B_ level–both of which are obtainable with relative ease without the involvement of complicated dynamic tests–demonstrated utility in discriminating between CHH and CDGP, and therefore could have the potential for wider application (10).

However, there seems to be consistent misapplication of “wait & see & reassure” guidance (intended for individuals with undifferentiated pubertal delay) to those with red flag markers of CHH who should, instead, be presumed hypogonadal until proven otherwise and receive sex hormone replacement from mean age of pubertal-onset in that population i.e., 12 years in boys (6, 15), whereas treatment should not be unduly withheld in those without CHH features but have turned 14 years old (16).

As a result, the diagnosis of CHH and initiation of clinically-meaningful treatment to induce puberty is typically unduly delayed until late adolescence or early adulthood. Despite different survey techniques across different European countries, data on the mean ages of diagnosis and initiation of clinically-meaningful^1^ treatment for CHH men have been remarkably consistent, with Dwyer et al (web-based, pan-European; *n* = 101) finding these to be 18 ± 6 and 19 ± 5 years, respectively (7); Raivio et al (nationwide Finnish cohort study) finding the median age of starting treatment to be 18.3 years (range, 11–34 years) (17), and Quinton (case notes based survey; *n* = 200) finding it to be 18.9 ± 9 years (18).

A surprisingly common pitfall is the failure to recognize the significance of cryptorchidism in an adolescent with pubertal delay. In retrospective CHH series, between 30 and 50% of CHH males have a history of cryptorchidism, of whom up to 2/3 have bilateral undescended testes (8, 19–21). By contrast, cryptorchidism is very rare in CDGP. In a large series of boys seen at a specialized center referred from primary care for delayed puberty, only 2% of CDGP boys had a history of cryptorchidism, compared with 36% of those with CHH (10). Hence, a history of cryptorchidism should alert clinicians to presume the diagnosis of CHH until proven otherwise (22). A concomitant family history of cryptorchidism, micropenis, infertility and/or non-reproductive features such as anosmia, renal agenesis and cleft palate/lip could provide valuable clues to an underlying diagnosis of CHH, though it is absent in majority of cases, in part due to variable disease penetrance and phenotypic expression, as well as oligogenic inheritance with unaffected parents (4).

Likewise, the significance of bilateral undescended testes in neonates as a possible indicator of CHH is typically underappreciated. Persistent bilateral cryptorchidism is found in a quarter of CHH infants, as compared to <0.7% among infants in the general population (Table 1) In a large single-center retrospective series, only one-third of CHH males with history of bilateral orchidopexy in childhood were referred to pediatric endocrinology for evaluation, with most cases re-presenting much later in life with absent puberty (19). Current clinical guidelines have largely focused on evaluation for possible congenital adrenal hyperplasia (CAH) and disorder of sex development (DSD), and neglected to provide sufficient guidance on the investigations necessary in cryptorchid boys to rule out CHH (36, 37), resulting in missed opportunity for early recognition.

**Table 1:** “Red Flag” clinical markers for congenital GnRH deficiency^*^.

**INDICATORS OF ABSENT MALE MINIPUBERTY**
**All apparent at or shortly after birth**
- Cryptorchidism (38% compared with general population prevalence 3.68–6.9% at birth, 1.0–2.4% at 3 months) (23–26) - Bilateral cryptorchidism (25% compared with general population 1.66–4.54% at birth, 0.09–0.66% at 3–12 months) (23–26) - Microphallus (9% compared with general population birth prevalence 0.015–0.35%) (27, 28) - Absent erections on nappy change
**NON-REPRODUCTIVE PHENOTYPES STRONGLY ASSOCIATED**
**WITH CHH**
**Apparent at or shortly after birth**
- Cleft lip and/or palate (5% compared with general population prevalence 0.1%) (29–31) - Hearing impairment *via automated optoacoustic emissions test* (6% compared with UK birth prevalence 0.12%) (32)
**Usually apparent by 6–8 years of age**
- Anosmia or hyposmia [43% compared with general population prevalence 1.4–19.1% (comprising both congenital & acquired)] (33–35)
**FAMILY HISTORY OF CHH**
Including offspring of CHH patients from ovulation- or spermatogenesis- induction (risk apparent even preconception)

* *Composite data including 4 published studies (8, 19–21)*.

As a result of late diagnosis and delayed treatment, the optimal standard of care is not delivered with patients with CHH, leading to significant psychosocial and reproductive sequelae.

## Unmet Health Needs

### Poor Psychological Well-Being

Psychological morbidities and antidepressant usage are highly prevalent among CHH men (17). In an international study comprising of participants from North and South America, Europe and Australasia, nearly two-thirds suffered from depression, with majority of them exhibiting moderate to severe symptoms (38). An important consequence arising from chronic affective disorders is poor adherence to long-term hormone replacement. It is concerning that more than one-third of the survey respondents reported long gaps (>1 year) in treatment, which could potentially exacerbate affective symptoms in turn as part of a vicious cycle.

The psychosexual impact of the disease is tremendous. A significant number of patients suffer from anxiety and low self-esteem, resulting in inability to form intimate relationships and social isolation. Consequently, in the aforementioned study cohort (38), approximately 50% of the men were not in a stable relationship at the time of survey, and many never had a sexual partner.

On the positive side, the majority of men surveyed had received tertiary level education and were in gainful employment, reflecting reasonable socioeconomic circumstances, although this did not negate the psychological effects of their disorder. However, CHH men with adopted or biological children were less likely to report depressive symptoms, suggesting the positive effect of family companionship, and the vicious circle that socially-withdrawn CHH men may be trapped in.

A major factor that contributes to poor mental health and impaired quality of life is the delay in diagnosis and the failure to administer age-appropriate induction of secondary sexual characteristic in many CHH males. As alluded to earlier, most patients only start to receive meaningful treatment in late adolescence (median age 18–19 years) typically after a prolonged and frustrating diagnostic odyssey (frequently shared by their female counterparts too) (1). Consequently, they are at risk of developing a multitude of psychosocial problems associated with pubertal delay, including low self-esteem, social withdrawal, poor school performance and even higher rates of substance use disorder (16, 39, 40). Furthermore, inadequately treated cryptorchidism and/or micropenis can lead to long-lasting adverse impact on their sexuality (17).

### Compromised Fertility Potential

In young adult males with CHH, gonadotrophin treatment achieves a significantly greater positive impact than direct testosterone (T) replacement on health-related quality of life (41). While both treatments are effective in improving physical function and general health, patients receiving gonadotrophins perform better in psychological domains, including emotional and mental health, particularly if sperms are found in ejaculate. This strongly suggests that patients psyches are deeply affected by their perceived chances of achieving paternity.

The infertility in CHH patients is due to spermatogenic failure, which is potentially amenable to GnRH or gonadotrophin treatment. Unfortunately, classic spermatogenic treatment–human chorionic gonadotrophin (hCG) monotherapy or combined gonadotropin treatment (hCG+FSH)–is much less successful in men with severe CHH (testes <4 mL), particularly those with history of bilateral cryptorchidism, than in men with HH of postpubertal-onset, e.g., due to acquired pituitary disease (42). Nonetheless, in centers experienced in the care of CHH patients, up to three-quarters can achieve spermatogenesis during hormonal induction treatment (6, 43), and pregnancy rates can be further enhanced with assisted reproductive techniques (6, 44).

Patients with rare medical disorders, defined by a prevalence of <5 in 10,000 in the population, often face challenges due to the lack of knowledge of their healthcare providers (45, 46). As such, specialized centers with expertise in diagnosis and interdisciplinary treatment of rare diseases are vital in the provision of care to these patients (47). Similarly, patients with CHH should ideally receive tertiary-level care to avoid gaps in treatment, and benefit from the latest technologies and advances in the research field. Early diagnosis would provide the opportunity for patients to receive appropriate and consistent care and support at specialized centers without delay. Treatment can also be tailored according to the needs and goals in different stages of life (6, 48).

But in real-life setting, according to one survey, it appears that only a minority of CHH men seeking fertility are able to achieve desired outcomes on fertility-inducing treatment (7). It is unclear how many of these men were treated at centers with the necessary expertise, but given that only half of the whole study cohort being followed-up at specialized/academic centers, access to such resources is likely to be correspondingly restricted.

### Elevated Risk for Low Bone Mineral Density

Chronic sex steroid deficiency is a major risk factor for osteoporosis and fragility fractures that affects both genders. As men with CHH have early-onset of T insufficiency, a delay in and/or lack of adequate androgen replacement would result in poor bone mass accretion and accelerated bone loss (49). Indeed, patients who are initiated on T replacement at older age appear to accrue less bone mineral compared to younger age, further supporting the importance of timely treatment (50). Nonetheless, even for those who are diagnosed and started on treatment only later in life, encouraging improvement in bone mineral density, particularly at trabecular-rich lumber spine, has been observed (51).

## Minipuberty–Critical Period of Genitalia Development and the Window to Early Diagnosis

High incidence of cryptorchidism and microphallus is observed in CHH males because of the absence of minipuberty, which is a critical period in the ontogenesis of the male reproductive tract, characterized by activation of the GnRH axis in the initial months postnatal. During this developmental phase, serum T and gonadotropin levels rise rapidly and peak at age 3 months–remarkably approaching adult male levels–before to mid-childhood quiescence by about 6 months of age (52, 53).

This robust hormonal activity is necessary to complete the processes of inguinoscrotal testicular descent and anchoring in the scrotum as well as penile growth, which had begun earlier *in utero* during the third trimester. Specifically, LH-stimulated secretion of T and INSL3 peptide by Leydig cells are the key factors involved in driving these physical changes (54, 55). There is a concurrent exponential increase in FSH-stimulated I_B_ and anti-Müllerian hormone (AMH) secretion, signifying active proliferation of Sertoli cells. Expansion of Sertoli and germ cells and seminiferous tubule formation are key determinants of future fertility potential and are responsible for 90% of testicular volume (56). Despite the brisk gonadotrophic activity and T secretion during this proliferative phase, germ cell maturation and spermatogenesis do not occur, because androgen receptors are not expressed on Sertoli cells until 5 years of age (57).

Following minipuberty, the hypothalamic-pituitary-testicular (HPT) axis retreats into quiescence for the rest of childhood. Serum T, LH, and FSH levels decline to low levels until reactivation of gonadal axis occurs in early adolescence, heralding the onset of puberty and marked by testicular enlargement (≥4 mL), followed by penile and pubic hair growth. At this stage, Sertoli cell maturation occurs, evidenced by a rise in I_B_ and decline in AMH levels, and spermatogenesis is achieved by the concerted actions of FSH and intra-testicular T (58). Therefore, pulsatile GnRH secretion in the neonatal period appears to be paramount for the normal development of male genitalia, with a far-reaching impact on male reproductive phenotype and fertility potential later in adult life.

Another important clinical implication of minipuberty is that it potentially provides a window-of-opportunity to facilitate detection of children with congenital GnRH deficiency, who would demonstrate abnormally low FSH, LH, and T levels if measured, thereby offering the advantage of a definitive prepubertal diagnosis and sign-posting them to pre-planned pubertal-induction with sex hormones at median age of pubertal-onset, rather than expectative management.

### Early Diagnosis for Avoiding Delay in Pubertal Induction

Diagnosis of CHH in early life facilitates the structuring of long-term surveillance and treatment plans, as well as ensuring that counseling and psychological support to patients and families are made available. When patients reach early adolescence, age-appropriate pubertal induction treatment will ensure that secondary sexual characteristics develop in tandem with peers (15), thereby avoiding the delay that has been traditionally experienced by most CHH men. As such, uncertainties are minimized and anxieties can be allayed.

### Potential Benefit of Early Diagnosis in Improving Prospect of Fertility

Early identification of boys with CHH could also plausibly provide the opportunity for intervention to optimize fertility potential. Although GnRH or gonadotrophin combination treatment are effective for most CHH men in spermatogenesis-induction, sperm outcomes are generally suboptimal (43, 59, 60). Moreover, nearly one-third with severe CHH remain azoospermic even with prolonged combined gonadotropin therapy, and hCG-monotherapy has proved to be despairingly ineffective.

Clinical features (consistent with long-standing severe GnRH deficiency minipuberty) that are predictive of poor treatment response, include: complete absence of puberty at presentation, cryptorchidism (especially if bilateral), low serum I_B_ concentrations (indicating depleted Sertoli cells) and prepubertal testicular volume (indicating depletion of Sertoli and germ cells and seminiferous tubules) (43, 59). On the other hand, CHH men with partial GnRH deficiency (testicular volume ≥4 mL) respond much better to combined gonadotrophin treatment, with around 80% achieving sperm in the ejaculate (61).

Therefore, in men with complete CHH, it would be theoretically advantageous to first maximize proliferation of Sertoli and germ cell and growth of seminiferous tubules by administering FSH-monotherapy prior to the introduction of hCG, so as to prevent premature maturation of a depleted pool of Sertoli cells under the influence of intra-testicular T.

Pathfinder studies of FSH-monotherapy in children and adolescents with HH of prepubertal-onset demonstrated its efficacy in promoting testicular growth and circulating I_B_ concentrations (62, 63). In particular, there was an encouraging spermatogenesis response in a subgroup of CHH adolescents, in whom FSH-priming before the combination with hCG successfully induced spermatogenesis in 3 out of 4 patients (63).

The potential benefit of unopposed FSH treatment was further studied in a randomized, open-label trial of 13 treatment-naive adult CHH men with prepubertal testes (<4 mL) (64). Seven men were randomized to recombinant FSH-pretreatment for 4 months before embarking on a 24-month GnRH treatment protocol. During the FSH-only phase, testicular volume doubled and I_B_ levels rose to adult levels, and all subjects in this arm subsequently developed sperm in ejaculate on GnRH therapy, compared to 4 of 6 men in the 24-month GnRH-only arm. There were also trends to larger testicular volume, higher maximal sperm counts and shorter time to first appearance of sperm in the ejaculate in the FSH-pretreated group.

Therefore, the findings of the benefits of FSH-priming and the potential deleterious effect of premature hCG therapy would be important to inform clinicians on the choice of treatment in adolescents with CHH, which would be greatly facilitated by timely diagnosis.

### Potential of Neonatal Gonadotrophin Treatment to Further Optimize Outcomes

Recognizing the critical role of minipuberty in the development of external genitalia and Sertoli cell proliferation and its subsequent influence on future reproductive function, the feasibility and benefits of recreating the physiological hormonal milieu in male CHH infants has been studied.

In the first published report of a boy with CHH and micropenis who received short-term recombinant human LH and FSH from age 7.9–13.7 months, the penile length successfully increased by 50% and the testicular volume nearly tripled by the end of treatment (65). In another report of 2 male infants, one case each of combined pituitary hormone deficiency (CPHD) and CHH, 6-month gonadotrophin combination therapy via subcutaneous pump infusion initiated at age 8 and 20 weeks, respectively, led to 4-fold increase in both penile length and testicular volume (66). More recently, 3–6 months of continuous subcutaneous infusion of recombinant human gonadotrophins in 5 male infants [4 CHH, 1 CPHD] produced several-fold increase in I_B_ concentrations, testicular volume, and T secretion (67).

Besides these encouraging responses to combined gonadotrophin treatment during infancy, it could also aid in the management of undescended testes. Cryptorchidism is present in approximately one-half of boys with severe CHH (19), and it is an independent predictor of infertility. Delay in orchidopexy has been associated with dramatic decline in germ cells (68), and as such it is generally recommended that surgical treatment take place by about 1 year of age (37, 69). However, small testes render surgical manipulation technically challenging and would result in excess risk of testicular trauma and tissue loss (70). By administering a period of presurgical gonadotrophin therapy, it allows the enlargement of testicular volume to facilitate the procedure.

Indeed, there are emerging evidence that spontaneous testicular descent could be successfully induced by gonadotrophin treatment in infants with central hypogonadism, and thus obviate the need for surgery. In a cohort of eight infants with maldescended testes due to underlying hypogonadotrophic hypogonadism (5 CHH, 3 CPHD), gonadotrophin infusion induced full testicular descent in 6 boys and partial descent in 2 boys, such that only 1 had to undergo orchidopexy nearly a year later because of re-ascent of both testes (71). Another study showed that combined recombinant FSH and LH in the first 6 months of life successfully induced spontaneous testicular descent in 2 of 4 bilateral-cryptorchid CHH/CPHD boys (67).

Therefore, short-term neonatal gonadotrophin treatment in ascertained cases of hypogonadotrophic hypogonadism appears to be effective in replicating the effects of minipuberty, by correcting micropenis, promoting testicular growth due to Sertoli cell expansion, and inducing spontaneous descent of malpositioned testes. Importantly, treatment was well-tolerated in all reported cases. Although definitive evidence is currently lacking, the prospect of early childhood hormonal intervention in CHH boys in augmenting sexual and reproductive function in adult life is worth serious consideration, and hopefully will provide impetus for larger clinical trials.

## A Proposed Strategy to Improve Detection of Male Infants With Severe CHH

The phase of male minipuberty provides an extraordinary useful diagnostic window to confirm (or refute) the diagnosis of congenital hypogonadism with a relatively straightforward biochemical hormone profiling without the need for complex dynamic testing. In a cohort of CPHD male infants (predominantly presenting with hypoglycaemia), findings of low circulating FSH, LH, and T concentrations reliably identified concomitant hypogonadotrophic hypogonadism in 14 of 15 infants with genitalia anomalies, whereas all other boys with normal genitalia demonstrated intact pituitary-gonadal axis function (72). This contrasts starkly with the conundrum of differentiating CHH from CDGP in adolescence.

However, unlike CPHD, neonates with CHH without cryptorchidism do not necessarily have clinical manifestations that would trigger referral to pediatric endocrinologists for pituitary hormonal evaluation. In addition, there is a lack of awareness and clinical guidance to consider CHH as a differential diagnosis of male infants with cryptorchidism, particularly if bilateral or associated with micropenis, so that appropriate evaluation can be undertaken (22, 37, 69). Indeed, the presence of “red flag” markers (Table 1) should warrant further investigations to exclude such a possibility.

Another challenge would be the interpretation of less robust serum gonadotrophin and testosterone results in the male infants. Of relevance, normative data of several important reproductive hormone values during minipuberty–including LH, FSH, T (by both radioimmunoassay and tandem mass spectrometry), AMH and I_B_-have recently been derived from a large cohort of healthy Danish infants, with cut-off values discriminating between sexes determined (53). By extension, values above the cut-off levels–generally lower than adult ranges–could be regarded as intactness of HPT axis function, whereas equivocal biochemical results that lie below the cut-off values may warrant repeat testing. Furthermore, expanding screening panel to include AMH and I_B_ could improve diagnostic confidence. Crucially, this study also demonstrated that the various sex hormones peak just before 3.5 months of age, suggesting that diagnostic performance would be most optimal when evaluation is undertaken around this time.

### Bilateral Cryptorchidism as Potential Screening Criterion

While routine screening for minipuberty is impractical, a targeted approach to evaluate male infants with suspicious signs of CHH could be cost-effective. Among the associated clinical features, bilateral cryptorchidism is particularly important because of its high prevalence (13.9–34.5%) among CHH males and the association with severe GnRH deficiency that has both prognostic and therapeutic implications (8, 19, 21).

Although cryptorchidism is a common congenital urogenital abnormality in newborns, most would undergo spontaneous descent in the absence of hypogonadism or other organic disorders. Data from prospective studies shows that the prevalence of bilateral cryptorchidism decreases substantially from 1.66 to 4.54% at time of birth to 0.09–0.66% by 3–12 months of age (23–26). In addition, spontaneous descent is unlikely to occur beyond 3 months postnatal (73), which coincides closely with the expected peak of minipuberty, making it an ideal time point to undertake testing of reproductive hormones in boys with persistent bilateral cryptorchidism. Moreover, although non-CHH cryptorchid boys may also exhibit hormonal abnormalities, they tend to have higher FSH, similar T and slightly lower I_B_ values compared to healthy infants, which are different from the biochemical pattern expected of in CHH male infants (74).

Therefore, testing male infants with bilateral cryptorchidism with or without micropenis at 3 months of age for absent minipuberty could potentially be a feasible approach to facilitate the early diagnosis of CHH. The diagnostic yield of such a selective screening strategy is explored here, using Britain's birth data as an example for mathematical illustration:

- With an average male live birth rate of 390,070 per annum in Britain (75), between 26 and 88 boys born each year could be affected by CHH (based on estimated prevalence of 1 in 4,415–15,000).
- On the other hand, bilateral cryptorchidism could affect between 6,475 and 17,709 (1.66–4.54%) of all boys at birth, regardless of the presence of CHH. Among them, between 4 and 30 infants could have underlying CHH (as bilateral cryptorchidism affects 13.9–34.5% of CHH population), thus representing 0.02–0.46% of all bilateral-cryptorchid boys at birth.
- By 3 months of age, following spontaneous testicular descent expected in majority of non-CHH infants, the total number of infants with persistent bilateral cryptorchidism would be expected to reduce substantially to between 351 and 2,574 (0.09–0.66%).
- As spontaneous testicular descent is not expected in CHH-affected infants, they now account for greater proportion of all persistently bilateral-cryptorchid boys: 0.16–8.55% (in contrast to 0.02–0.46% at birth). Hence, screening at 3 months of age appears to be most cost-effective by avoiding investigations in vast majority of infants without persistent bilateral cryptorchidism.

Based on this hypothesis, possibly up to 1 in 11–12 male infants with persistent bilateral cryptorchidism at 3 months of age could have underlying CHH. Interestingly, this estimate is consistent with the findings from an historical surgical series of patients who had been treated with orchidopexy. In this study, of the 98 patients evaluated for possible underlying endocrine cause of cryptorchidism, 2 were found to have CHH in adult life and both of them had bilateral undescended testes (76). That represents 6.1% (2/33) of all individuals with bilateral cryptorchidism in the series.

However, it is worth reiterating that, at present, there is no literature to suggest that CHH screening in infancy has been systematically studied by any research group, and thus is a working concept that remains in early exploratory phase. Further long-term multicentre research is necessary to validate the utility of any screening strategy that seeks to establish early diagnosis of this rare condition, as well as the effectiveness and safety of hormonal treatment in CHH infants. Protocols should also be developed collaboratively by pediatric and adult endocrinologists to ensure that these children are placed in a structured follow-up and transition programme, thereby ensuring that no one falls through the crack in the health system.

### A Short Note on Pre-pubertal Acquired Hypogonadotrophic Hypogonadism

Pubertal failure in male adolescents with known history of acquired hypopituitarism generally avoids the diagnostic conundrum of CHH, hence allowing pubertal-induction therapy to be planned for at around 12 years of age, with gradual increase in testosterone dose before reaching adult replacement dose in about 3 years (15). Importantly, because of preserved minipuberty, normal Sertoli cell proliferation is expected in early childhood, and testicular maldescent would be less likely to occur (63). Therefore, these individuals tend to have a more optimistic fertility outlook, with greater spermatogenic response to gonadotrophin treatment anticipated (77), unless testicular tissue has been compromised e.g., prior gonadotoxic chemotherapy treatment.

## Conclusion

Early detection of CHH through detection of absent minipuberty can potentially modify patients' experience by facilitating timely interventions at different stages of life. This is achievable with a systematic approach in place to identify male infants with “red flag” markers of CHH, particularly bilateral cryptorchidism, so that biochemical testing can be undertaken within the narrow diagnostic window.

Neonatal gonadotrophin treatment appears to be beneficial in correcting micropenis and even cryptorchidism. In adolescents, age-appropriate pubertal induction is the main goal, and a brief course of FSH monotherapy should be considered to optimize future fertility potential. Close collaborations between pediatric care providers and adult endocrinologists would ensure that these patients transit smoothly to adulthood, during which the aims of treatment would shift to fertility-induction and long-term androgen replacement. Equally important are the psychological support and genetic counseling that should be provided along the way to empower patients, and families, so that they can cope with their conditions confidently.

## Future Directions

Proactive screening of male infants with bilateral cryptorchidism (and/or micropenis) is anticipated to have a direct impact on the quality of care delivered to affected children by establishing early diagnosis. However, this issue has not been addressed by major clinical guidelines developed for the management of infants with abnormal genitalia, thereby leading to missed opportunities for patients and clinicians alike in accessing the most appropriate treatment possible. To overcome this barrier, it is imperative that collaborative research by key stakeholder centers seek to clarify the feasibility of such an approach systematically, as well as expanding the work on neonatal gonadotrophin treatment, so as to advance the agenda for wider adoption among endocrinologists, pediatricians, and pediatric surgeons.

## Author Contributions

All authors listed have made a substantial, direct and intellectual contribution to the work, and approved it for publication.

### Conflict of Interest Statement

The authors declare that the research was conducted in the absence of any commercial or financial relationships that could be construed as a potential conflict of interest.

## Footnotes

^1^For instance, 50mg intramuscular testosterone monthly would not remotely constitute appropriate or meaningful replacement therapy for a 20 year-old, 1.7m and 70kg prepubertal male with Kallmann syndrome.
